# Trace Level Quantification of the (−)2-(2-amino-5-chlorophenyl)-4-cyclopropyl-1,1,1-trifluoro-3-butyn-2-ol Genotoxic Impurity in Efavirenz Drug Substance and Drug Product Using LC–MS/MS

**DOI:** 10.3390/scipharm84030456

**Published:** 2015-10-18

**Authors:** Nagadeep Jaishetty, Kamaraj Palanisamy, Arthanareeswari Maruthapillai, Rajamanohar Jaishetty

**Affiliations:** 1 Department of chemistry, SRM University, Kattankulathur, Channai-603203, Tamilnadu, India; kamaraj97@yahoo.co.in (K.P.); arthanareeswarim@gmail.com (A.M.); 2 Department of chemistry, Osmania University, Hyderabad, Telanagana-500007, India; raja_manohar@hotmail.com

**Keywords:** LC–MS/MS, genotoxicity, method development, efavirenz, validation

## Abstract

Efavirenz is a non-nucleoside reverse transcriptase inhibitor used in the treatment of human immunodeficiency virus type-1 (HIV). (2S)-(2-Amino-5-chlorophenyl)-4-cyclopropyl-1,1,1-trifluoro-3-butyn-2-ol (AMCOL), used as an intermediate in the synthesis of efavirenz and a degradation impurity, has an aminoaryl derivative which is a well-known alerting function for genotoxic activity. Upon request from a regulatory agency, a selective and sensitive liquid chromatography–tandem mass spectrometry (LC–MS/MS) method was developed for trace level quantitative determination of AMCOL related compound of efavirenz, for a risk assessment and comparison of impurity levels with the commercially available innovator product (brand name: Sustiva). The method provided excellent sensitivity at a typical target analyte level of <2.5 ppm, an established threshold of toxicological concern (TTC), when the drug substance and drug product samples were prepared at 15.0 mg/mL. The AMCOL sample was analyzed on a Luna C18 (2) (100 mm × 4.6 mm, 3 µm) column interfaced with a triple quadrupole tandem mass spectrometer operated in a multiple reaction monitoring (MRM) mode. Positive electrospray ionization (ESI) was employed as the ionization source and the mobile phase used was 5.0 mM ammonium acetate-methanol (35:65, v/v). The calibration curve showed good linearity over the concentration range of 0.2–5.0 ppm with a correlation coefficient of >0.999. The limit of detection (LOD) and limit of quantification (LOQ) were found to be 0.07 and 0.2 ppm, respectively. The developed method was validated as per international council on harmonization (ICH) guidelines in terms of LOD, LOQ, linearity, precision, accuracy, specificity, and robustness.

## 1. Introduction

**Figure 1:**
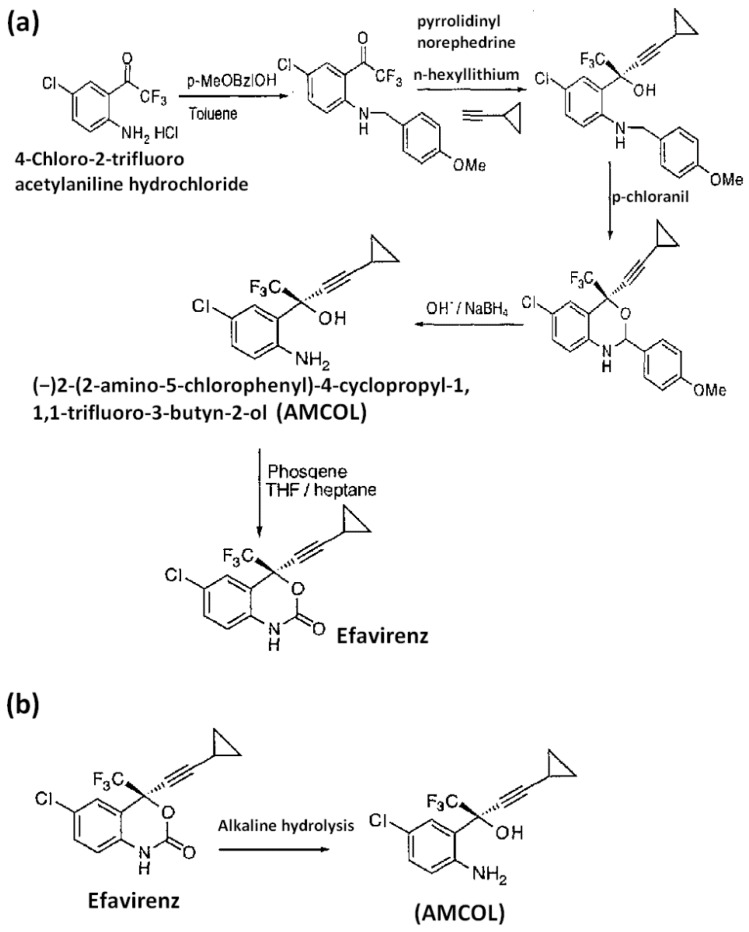
Schematic reaction mechanism showing the formation of (**a**) (2S)-(2-Amino-5-chlorophenyl)-4-cyclopropyl-1,1,1-trifluoro-3-butyn-2-ol (AMCOL) during the synthesis of efavirenz and (**b**) degradation of efavirenz to AMCOL.

Chemically, efavirenz is (−)6-chloro-4-cyclopropylethynyl-4-trifluoromethyl-1,4-dihydro-2H-3,1-benzoxazin-2-one. The efavirenz drug is administrated for the treatment of human immunodeficiency virus (HIV) to slow down damage to the immune system and prevent the occurrence of acquired immunodeficiency syndrome (AIDS)-defining illness. The drug is used in combination with other antiretroviral agents for the treatment of HIV-1 infection in children and adults. The usual dose of efavirenz is 600 mg per day. Efavirenz was developed by Du Pont Pharma (Wilmington, DE, USA) and it was approved for HIV treatment in the United States in 1998 and the European license was granted in May 1999. Efavirenz is marketed by Bristol-Myers Squibb (New York, NY, USA) under the trade name Sustiva in the United States, Australia, United Kingdom, and other European countries. Generic versions of efavirenz tablets are manufactured in India and other countries by different manufacturers.

(2S)-(2-Amino-5-chlorophenyl)-4-cyclopropyl-1,1,1-trifluoro-3-butyn-2-ol (AMCOL) is the most important intermediate during the synthesis of efavirenz, given in Figure 1a [1], and has an aminoaryl derivative, a well-known alerting function for genotoxic activity. Hence, it is identified as a potential genotoxic impurity (PGI) in efavirenz [2,3]. However, no methods have been reported for trace level determination of AMCOL, a process-related and degradation impurity of efavirenz (Figure 1b).

Literature survey revealed that there are analytical methods available for the determination of efavirenz from biological matrices [4,5,6,7], and few methods reporting efavirenz impurity analysis have been described in the literature [8,9,10,11,12] and pharmacopeia [13,14,15]. The reported methods were not suitable for the desired low level (<1.5 µg/day) quantification of the AMCOL impurity for a risk assessment and comparison with the impurity levels of the commercially available innovator product (brand name: Sustiva). In the last few years, growing importance has been given to the quantification of potential genotoxic impurities [16,17,18,19], i.e., those which could cause DNA damage involving genetic mutations [20]. According to the current regulatory guidance for genotoxic impurities [21,22], analytical methods should be developed to meet the required intake limit of 1.5 µg/day of the individual impurity. Based on the threshold of toxicological concern (TTC) limit of 1.5 µg/day and on the maximum adult daily dose of efavirenz of 600 mg/person, its genotoxic impurities are required to be controlled at a concentration limit of 2.5 µg/g (ppm) in the drug substance and drug product. Due to its higher sensitivity and selectivity, liquid chromatography–tandem mass spectrophotometry (LC–MS/MS) has been applied for the quantification of AMCOL, a process-related and degradation impurity in the drug substance and drug product. The developed method was fully validated as per international council on harmonization (ICH) guidelines in terms of limit of detection (LOD), limit of quantification (LOQ), linearity, precision, accuracy, specificity, and robustness.

## 2. Materials and Methods

### 2.1. Reagents and Standards

High-performance liquid chromatography (HPLC)-grade acetonitrile, methanol, and ammonium acetate were purchased from Merck (Mumbai, India). Acetic acid was obtained in analytical grade from SD Fine Chemicals Limited (Mumbai, India). Purified water was collected through the Milli-Q-Plus water purification system (Millipore, Milford, MA, USA). The reference substance of AMCOL was obtained from Sigma-Aldrich (St. Louis, MO, USA). Efavirenz was a gift sample from a local manufacturing company in Chennai, India. Polyvinylidene difluoride (PVDF) filter membranes (0.22 µm × 47 mm diametre) were purchased from Fisher Scientific Pvt. Ltd., (Mumbai, India).

### 2.2. Preparation of Buffer Solution

An amount of 0.39 g of accurately weighed ammonium acetate was diluted to 1000 mL with Milli-Q water, its pH was adjusted to 6.5 ± 0.05 with diluted acetic acid, and it was filtered through a 0.22 µm nylon membrane filter paper. The mobile phase solution consisting of ammonium acetate and methanol (35%:65% (v/v) at pH 6.5 ± 0.05) was prepared and filtered through a 0.22 µm nylon membrane sample filter paper and degassed. All the solutions were stored at ambient temperature.

### 2.3. Preparation of Standard and Sample Solutions

A stock solution of efavirenz (15.0 mg/mL) was prepared by dissolving an appropriate amount in acetonitrile and a stock solution of AMCOL at 0.15 mg/mL was also prepared in acetonitrile. The diluted stock solution (0.0075 mg/mL) was prepared by diluting 5.0 mL of the 0.15 mg/mL solution to 100 mL with acetonitrile. The working standard solution was prepared by dissolving 150 mg of accurately weighed efavirenz into a 10 mL volumetric flask and the solution was made up to the graduation mark after adding 5.0 µL of 0.075 µg/mL diluted stock solution to give 37.5 ng/mL and 15.0 mg/mL of PGIs with respect to efavirenz, which corresponds to 2.5 ppm of AMCOL contamination relative to the drug substance. The GTI samples for validation at 0.2, 1.25, 2.5, and 5.0 ppm concentrations relative to the drug substance were prepared in the same manner using 0.075 µg/mL of diluted stock solution. The concentrations of the standard solutions and samples were optimized to achieve a desired signal-to-noise ratio (S/N) and good peak shape. All the standards were sonicated for 10 min and then filtered through 0.22 µm membrane filters before analysis.

### 2.4. Instrumentation

The mass spectrometry (MS) system used was an Applied Biosystems Sciex API 4000 model (Zug, Switzerland) and was coupled with the HPLC system consisting of an LC-20AD binary gradient pump, SPD-10AVP UV detector, SIL-10HTC autosampler, and a column oven CTO-10ASVP (Shimadzu Corporation, Kyoto, Japan).

Data acquisition and processing were conducted using Analyst 1.5.1 software on a Dell computer (Digital equipment Co., Maynard, MA, USA).

### 2.5. Operating Conditions of Liquid Chromatography–Tandem Mass Spectrometry

**Figure 2:**
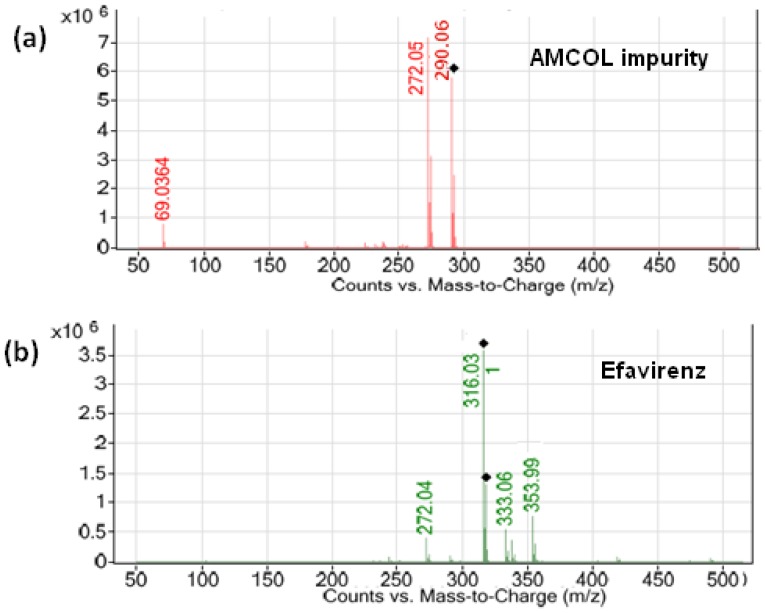
Typical mass spectrum scans of the (**a**) AMCOL impurity and (**b**) efavirenz.

The analytical column used in liquid chromatography–tandem mass spectrometry (LC–MS/MS) was the Luna C18 (2) (100 mm × 4.6 mm, 3.0 µm) column (Phenomenex Co., Torrance, CA, USA) in isocratic mode using 5.0 mM ammonium acetate-methanol in the ratio of 35:65 (v/v). The flow rate was 0.8 mL/min, which split down to 0.2 mL/min in the MS source. The column oven temperature was maintained at 45 °C, and the sample cooler temperature was set to 10°C. The injection volume was 10 µL. The positive electrospray ionization (ESI) probe operated with multiple reaction monitoring mode was used for the quantification of AMCOL. In this method, AMCOL was monitored with its transition ion pair *m/z* 290.2/272.1 (protonated). The ion spray voltage (V), declustering potential (DP), and entrance potential (EP) were kept as 5500 V, 50 V, and 10 V, respectively. The curtain gas flow, ion source gas 1, and ion source gas 2 nebulisation pressures were maintained as 15 psi, 18 psi, and 20 psi, respectively. All the parameters of LC and MS were controlled by Analyst software version 1.5.1. The typical mass spectrum scan for efavirenz and AMCOL are given in Figure 2.

### 2.6. Validation Study

The developed method was validated in terms of specificity, linearity, limit of quantification (LOQ), limit of detection (LOD), accuracy, precision, robustness, and solution stability. A thorough and complete method validation for the analysis of AMCOL was done according to international guidelines [23]. The method validation started by injecting 2.5 ppm of individual solutions of AMCOL with respect to 15.0 mg/mL of efavirenz, and its S/N ratios were determined. Then, to determine the LOD and LOQ values, the AMCOL concentration was reduced sequentially such that it yielded S/N ratios of 3:1 and 10:1, respectively. The precision of the LOD and LOQ values was experimentally verified by injecting six standard solutions of the compounds at the determined concentrations. Linearity for AMCOL was fixed in the range of the LOQ to 200% (0.2–5.0 ppm) of the estimated permitted level (viz. 2.5 ppm with respect to 15.0 mg/mL of efavirenz solution). Hence, 25%, 50%, 75%, 100%, 150%, and 200% solutions of AMCOL were prepared and injected individually while considering the 100% level as 2.5 ppm. The calibration curve was drawn between the peak areas versus concentration of AMCOL. The slope, intercept, and correlation coefficient values were derived from linear least-square regression analysis. The precision was evaluated at two levels, viz. repeatability and intermediate precision. Repeatability was checked by calculating the relative standard deviation (%RSD) of six replicate determinations by injecting six freshly prepared solutions containing 2.5 ppm of AMCOL on the same day. The same experiments were done on six different days for evaluating intermediate precision. To determine the accuracy of the method, a known amount of the sample was taken separately at different intervals and spiked with a known quantity of AMCOL (LOQ to 200% level). A recovery study by the standard addition method was performed to evaluate accuracy and specificity. Accordingly, the accuracy of the method was determined by spiking 0.2 ppm, 1.25 ppm, 2.5 ppm, and 5.0 ppm of AMCOL separately to three batches of the pure sample and three batches of formulation samples of efavirenz (15.0 mg/mL). Each determination was carried out three times. The specificity, defined as the ability of the method to measure the analyte specifically in the sample matrix, was determined by analyzing the tablets of efavirenz. The robustness of the method was studied with deliberate modifications in the flow rate of the mobile phase and column temperature. The optimized flow rate of the mobile phase was 0.9 mL/min and the same was altered by 0.1 units, i.e., from 0.8 mL/min to 1.0 mL/min. The effect of column temperature on resolution was studied at 48 °C and 42 °C instead of the method temperature (45 °C). However, the mobile phase components were held constant as described above. The stability of AMCOL in the diluent was checked by keeping it in an autosampler and observing the variations in its peak areas. Acetonitrile and water in the ratio 1:1 was tried as diluent in the sample preparation.

## 3. Results and Discussion

### 3.1. Method Development

The main aim of the present LC–MS/MS method was separation and quantification of AMCOL in the efavirenz active drug substance and drug product. Sample preparation is an important step in pharmaceutical impurity analysis to control matrix effects and to improve the sensitivity as well as achieve better analyte recovery. Several diluents like a mixture of methanol and tetrahydrofuran with water in different compositions were evaluated with respect to chromatographic efficiency and finally, we found that acetonitrile and water in the ratio 1:1 was a suitable solvent which provided good response, recovery, and proper peak shapes for AMCOL and efavirenz. Several attempts were made with different stationary phases including Kromasil C18, Luna C18 (2), Symmetry C18, and Zorbax Rx C8 (with different dimensions) to achieve proper separation of the analyte and drug substance. Kromasil C18 and Zorbax Rx C8 were not found to be suitable as the response of the analyte was found to be less and it was not well-resolved from the efavirenz drug substance peak. Only the symmetry C18 (100 mm × 4.6 mm, 3.5 µm) and Luna C18 (2) (100 mm × 4.6 mm, 3.0 µm) columns provided superior peak shape, baseline separation, desired linearity, and reproducibility. Different compositions of mobile phase using 5.0 mM ammonium acetate and methanol (10:90, 20:80 and 50:50, v/v) were checked for peak separation and sensitivity. Good separation and responses were observed using a mixture of 5.0 mM ammonium acetate–methanol (35:65, v/v). Both isocratic and gradient elution modes were evaluated, but the isocratic elution was observed to be more efficient in achieving optimum separation of the analyte from the drug substance peak. The column was thermostated at 45 °C to avoid any shift in retention time and for good peak shape.

**Figure 3:**
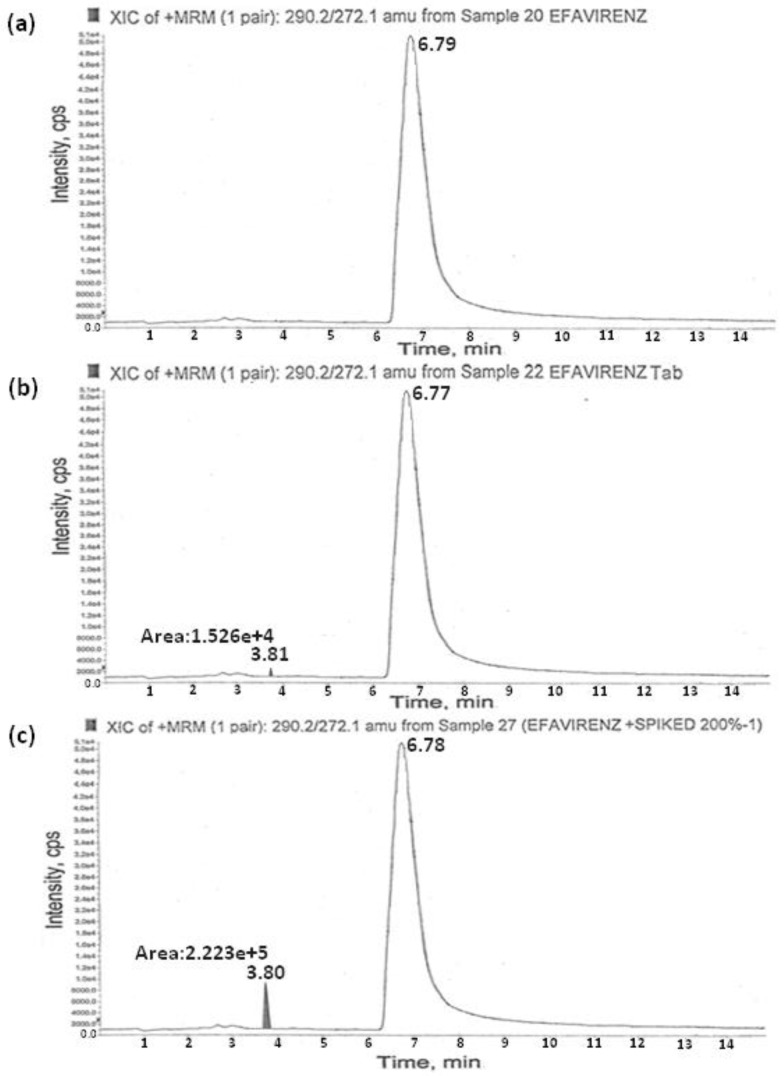
Typical chromatograms of the (**a**) efavirenz drug substance (**b**), efavirenz tablet, and (**c**) accuracy of AMCOL.

The signal intensity obtained for AMCOL in positive mode was much higher than that in negative mode. Then, the possibility of using electrospray ionization or atmospheric pressure chemical ionization (APCI) sources under positive ion detection mode was evaluated during the early stages of method development. The ESI spectra revealed higher signals for the molecule compared to the APCI source. Therefore, the method development was further limited to the ESI source. Retention times for AMCOL and efavirenz were observed to be about 3.82 and 6.7 min, respectively. The reproducibility of retention times for the analytes was expressed as % CV which was found to be less than 2.0% for 100 injections on the same column. Typical chromatograms of the drug substance and drug product are depicted in Figure 3.

### 3.2. Method Validation

The established method for the determination of AMCOL in efavirenz was validated according to US Food and Drug Administration (FDA) and ICH guidelines.

#### 3.2.1. Specificity

**Figure 4:**
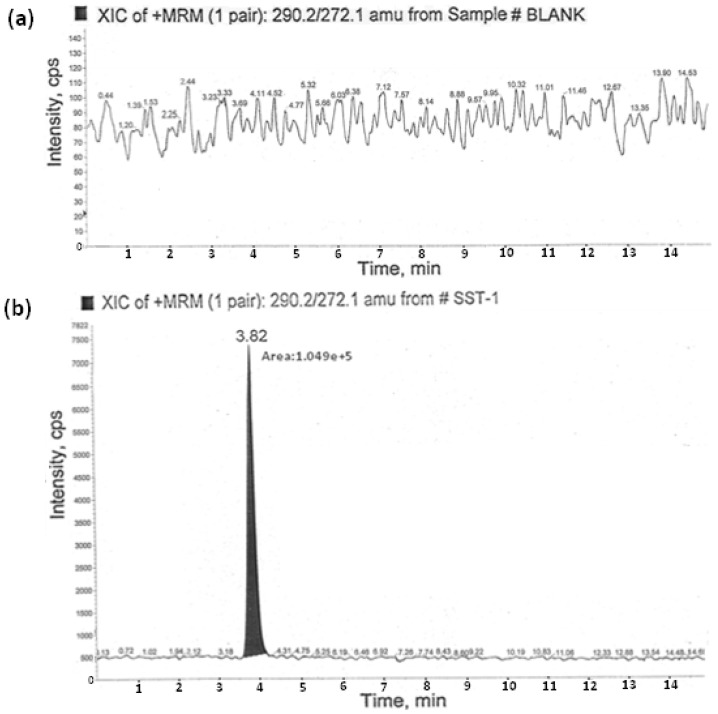
Blank chromatogram (**a**) and typical chromatogram (**b**) of AMCOL at the 2.5 ppm level. XIC: Extracted ion chromatogram; MRM: Multiple reaction monitoring; amu: atomic mass unit; SST-1: System suitability solution-1.

Specificity is the ability to assess the analyte unequivocally in the presence of the components which may be expected to be present in the sample matrix. The specificity of the method was best determined by analyzing the tablets of efavirenz. The solutions of efavirenz and AMCOL were prepared individually at specification levels in the diluent and the solution of efavirenz spiked with AMCOL was also prepared and injected into the LC–MS/MS. From the results we observed that the common excipients used in the tablets did not interfere at the retention times of the AMCOL impurity. The corresponding blank (acetonitrile and water in 1:1) and AMCOL standard chromatogram is shown in Figure 4.

#### 3.2.2. Precision

The precision of the method was evaluated at two levels, viz. repeatability and intermediate precision. Repeatability was checked by calculating the % relative standard deviation (%RSD) of six replicate determinations by injecting six freshly prepared solutions containing 2.5 ppm of AMCOL on the same day. The same experiments were done on six different days to evaluate intermediate precision. The developed method was found to be precise as the % RSD values for intraday precision were less than 1.0%, similarly the %RSD values for interday precision were found to be less than 2.0% (Table 1).

#### 3.2.3. Linearity

Linearity of the developed method was satisfactorily demonstrated with a seven-point calibration graph in the range of LOQ-200% of the estimated permitted level (viz. 2.5 ppm with respect to 15.0 mg/mL of efavirenz solution). The calibration curve was drawn between the peak areas versus the concentration of the analyte. The slope, intercept, and correlation coefficient values were derived from least squares linear regression analysis. The correlation coefficient obtained for AMCOL was >0.999 (Table 1). The linearity experiment revealed that the mass spectrometric responses were proportional to their concentration within the range of 0.2–5.0 ppm for the AMCOL impurity.

#### 3.2.4. Limit of Detection (LOD) and Limit of Quantification (LOQ)

To determine the LOD and LOQ values, we first injected a 2.5 ppm solution of AMCOL with respect to 15.0 mg/mL of efavirenz, and then the concentration was reduced sequentially to yield a S/N ratio of 3:1 and 10:1, respectively. Each predicted concentration was verified for its precision by preparing the solutions at about the predicted concentration and injecting each solution six times for analysis. The correlation coefficient obtained in each case was >0.9997. The predicted concentrations for the LOD and LOQ were 0.071 and 0.213 ppm, respectively. The results are given in Table 2.

#### 3.2.5. Recovery Studies

The recovery of the method was determined in triplicate at the LOQ, 1.25 ppm, 2.5 ppm, and 5.0 ppm concentrations in three batches of pure and formulation samples of efavirenz. The recovery data are presented in Table 3. The results exhibited excellent recoveries of AMCOL within the range of 96.8%–101.4%. Typical chromatograms of the spiked samples are given in Figure 3c.

#### 3.2.6. Robustness

Robustness of the method was determined by making deliberate changes in experimental conditions including flow rate and column oven temperature. The actual flow rate of the mobile phase was 0.9 mL/min and the same was altered by 0.1 units, i.e., 0.8 mL/min and 1.0 mL/min. The effect of temperature on chromatographic resolution was also studied at 48 °C and 42 °C (altered by 3 °C units). No significant change in the chromatographic performance was observed for all of the above deliberately varied experimental conditions, which indicated the robustness of the method.

#### 3.2.7. Stability of Solution

The stability experiments were performed thoroughly to evaluate the stability of AMCOL stock solutions at room temperature. The results demonstrated that the stock solution of AMCOL was stable at room temperature for 48 h. The values for the percent change for the above stability experiments are compiled in Table 4. The percentage recoveries of stock solution at different time intervals were within the range from 97.6% to 100.9% of their nominal values. The results obtained were then compared with the precision results of the method. The difference between recoveries at hours 0 and 48 was not more than 10%, which indicates that the sample prepared in diluent was stable for at least 48 h. Therefore, it is recommended to complete the analysis before 48 h of its preparation in acetonitrile and water (1:1).

## 4. Conclusions

The desired goal of the study was to develop a simple analytical method that is capable of quantifying AMCOL in the efavirenz drug substance as well as in the formulation sample. Hence, a simple LC–MS/MS method capable of quantifying the AMCOL impurity at permitted levels was developed and validated. The method was fully validated and presents good linearity, specificity, accuracy, precision, and robustness. The LOD and LOQ values for AMCOL were very low as 0.071 and 0.213 ppm, respectively. The sample prepared in analytical solution was found to be stable for at least 48 h. Therefore, the above-mentioned LC–MS/MS method for the analysis of the AMCOL impurity was found to be simple, selective, and sensitive. The method presented here could be very useful for monitoring AMCOL in efavirenz at trace levels during its manufacture and stability tests.

## Figures and Tables

**Table 1:** Validation Summary of AMCOL

Parameter	AMCOL
*Regression equation*	
Slope	41915.8
Intercept	1066.8
R value	0.9999
R^2^ value	0.9997
LOD (in ppm)	0.071
LOQ (in ppm)	0.213
Precision at LOQ *n* = 6 (% RSD)	2.3
Precision *n* = 6 (% RSD)	0.8
Intermediate *n* = 6 precision (% RSD)	0.6

LOD: Limit of detection; LOQ: Limit of quantification.

**Table 2:** LOD and LOQ Data of AMCOL

Injection ID	LOD (0.07 ppm)	LOQ (0.21 ppm)
1	2684	8568
2	2962	8865
3	2851	9014
4	2762	8465
5	2914	8856
6	2645	8752
Mean Area	2803.0	8753.3
Standard deviation.	127.1	204.2
% RSD	4.53	2.33

RSD: Relative standard deviation.

**Table 3:** Evaluation of Accuracy

Sample	% Recovery of AMCOL ^a^			
	0.2 ppm (LOQ)	1.25 ppm	2.5 ppm	5 ppm
Pure sample-I	96.8 ± 2.8	98.5 ± 0.8	99.6 ± 1.2	97.8 ± 2.1
Pure sample-II	100.3 ± 1.2	98.2 ± 0.6	98.6 ± 0.8	98.3 ± 1.1
Pure sample-III	98.1 ± 1.9	101.4 ± 1.2	98.3 ± 0.9	99.2 ± 1.2
Formulation sample-I	98.8 ± 1.2	97.5 ± 1.1	98.7 ± 1.1	99.8 ± 1.3
Formulation sample-II	101.3 ± 2.1	98.2 ± 0.6	100.2 ± 0.8	98.3 ± 1.1
Formulation sample-III	99.5 ± 2.4	100.6 ± 1.2	99.3 ± 0.9	98.2 ± 1.6

^a^ Mean ± %RSD for three determinations.

**Table 4:** Solution Stability Data of AMOCL at the LOQ Concentration

Sample Name	Time (h)	Peak Area	Theoretical Concentration (ppm)	Measured Concentration (ppm)	% Recovery
Pure sample	0 h	8753	0.213	0.209	98.1
	12 h	8810	0.213	0.211	99.1
	24 h	8713	0.213	0.208	97.6
	48 h	8816	0.213	0.214	100.5
Formulation sample	0 h	8865	0.213	0.212	99.5
	12 h	8923	0.213	0.213	100.3
	24 h	8710	0.213	0.211	99.1
	48 h	8689	0.213	0.215	100.9
